# The Type III Effector XopL*_Xcc_* in *Xanthomonas campestris* pv. *campestris* Targets the Proton Pump Interactor 1 and Suppresses Innate Immunity in *Arabidopsis*

**DOI:** 10.3390/ijms25179175

**Published:** 2024-08-23

**Authors:** Jing Huang, Yuru Dong, Nana Li, Yongqiang He, Hao Zhou

**Affiliations:** 1 State Key Laboratory for Conservation and Utilization of Subtropical Agro-Bioresources, Guangxi University, Nanning 530005, China; jhuang@gxu.edu.cn (J.H.);; 2 Guangxi Key Laboratory for Polysaccharide Materials and Modifications, Guangxi Minzu University, Nanning 530006, China

**Keywords:** *Xanthomonas campestris* pv. *campestris*, type III effector, XopL*_Xcc_*, *Arabidopsis thaliana*, innate immunity, proton pump interactor isoform 1

## Abstract

*Xanthomonas campestris* pathovar *campestris* (*Xcc*) is a significant phytopathogen causing black rot disease in crucifers. *Xcc* injects a variety of type III effectors (T3Es) into the host cell to assist infection or propagation. A number of T3Es inhibit plant immunity, but the biochemical basis for a vast majority of them remains unknown. Previous research has revealed that the evolutionarily conserved XopL-family effector XopL*_Xcc_* inhibits plant immunity, although the underlying mechanisms remain incompletely elucidated. In this study, we identified proton pump interactor (PPI1) as a specific virulence target of XopL*_Xcc_* in *Arabidopsis*. Notably, the C-terminus of PPI1 and the Leucine-rich repeat (LRR) domains of XopL*_Xcc_* are pivotal for facilitating this interaction. Our findings indicate that PPI1 plays a role in the immune response of *Arabidopsis* to *Xcc*. These results propose a model in which XopL*_Xcc_* binds to PPI1, disrupting the early defense responses activated in *Arabidopsis* during *Xcc* infection and providing valuable insights into potential strategies for regulating plasma membrane (PM) H^+^-ATPase activity during infection. These novel insights enhance our understanding of the pathogenic mechanisms of T3Es and contribute to the development of effective strategies for controlling bacterial diseases.

## 1. Introduction

*Xanthomonas* is a genus of Gram-negative phytopathogens that threatens >400 plant species worldwide. Most *Xanthomonas* species utilize the type III secretion system to directly inject type III effector proteins (T3Es) into plant cells [1]. Once inside, T3SEs contribute to pathogenesis, where a few are required for full pathogen virulence, and promote pathogen propagation in the host. Some are perceived by pattern recognition receptors (PPRs) to suppress pathogen-associated molecular pattern (PAMP)-triggered immunity (PTI); and others are monitored by the proteins of the host to activate strong defense responses [1,2,3].

*Xanthomonas campestris* pathovar *campestris* (*Xcc*) is the causal agent of black rot diseases in numerous crucifer plant genera, such as *Brassica* and *Arabidopsis* [4]. Recently, the pathogenesis mechanisms of *Xcc* have been studied widely, with >100 genes contributing to its pathogenicity [5,6,7,8,9]. In the *Xcc* 8004 genome, 34 putative genes encode T3Es [5]; yet, only a handful, including *XopD*, *AvrXccC, XopL*, *XopAC*, *XopAM*, *XopN*, and *XopJ*, have been functionally investigated [10,11,12,13,14,15,16,17,18]. Many of them inhibit plant immunity, but the underlying mechanisms are not fully understood.

XopL*_Xcc_* (also known as XopXccLR and XopL*_Xcc_*_8004_) is an LRR protein encoded by *XC_4273* (Gene ID: 3379891) [16,19,20]. Its homologs or analogs are present in all the sequenced *Xanthomonas* species or pathovars. XopLs play a significant role in the virulence of *Xanthomonas euvesicatoria* (*Xe*) strain 85-10 in tomatoes [21]; *X*. *axonopodis* pv. *punicae* (*Xap*) in pomegranates [22]; and *Xcc* 8004 in Chinese radish, Chinese cabbage, and *Arabidopsis* [16,19,20]. XopL*_Xcc_* is a crucial T3E that disrupts innate immunity in *Arabidopsis* by suppressing PTI signaling independent of mitogen-activated protein kinases (MAPKs) [16,20]. Despite these findings, the specific virulence targets and underlying mechanisms of XopL*_Xcc_* remain incompletely elucidated.

In plants, the plasma membrane (PM) H^+^-ATPase, the well-known PM H^+^ pump, is a central regulator in plant physiology, which mediates not only growth and development but also adaptation to diverse environmental stimuli [23,24]. In vivo, its activity is modulated by various signals, with the major regulators being 14-3-3 family proteins, which bind to the auto inhibitory domain in the C-terminus of the ATPase, thereby stimulating pump activity [25]. Limited information exists regarding the regulation of PM H^+^ ATPase by other effectors. Proton pump interactor 1 (PPI1) is a regulatory protein that interacts with the regulatory C-terminus of the *Arabidopsis* PM H^+^-ATPase at a site distinct from the 14-3-3 binding site, thereby stimulating its activity in vitro [24,26]. The main part of PPI1 is localized at the endoplasmic reticulum, from which it might translocate to the PM for interaction with H^+^-ATPase in response to as-yet-unidentified signals [27]. PPI1 is highly expressed in most plant organs [28] and has been documented in several species, including *Arabidopsis* [24], rice [29], potato, and tomato [30]. Additionally, previous research has revealed that PPI1 in plants responds to multiple abiotic stresses, including cold, salt, drought, and Fe deficiency stress [30,31]. However, its role in the plant immune response to pathogens remains unclear.

This study revealed that XopL*_Xcc_* enhances virulence and suppresses innate immunity by targeting the proton pump interactor 1 (PPI1), a potential player in *Arabidopsis* immune responses. Moreover, the C-terminus of PPI1 and the LRR domains of XopL*_Xcc_* play crucial roles in facilitating this interaction. These results led us to propose a model in which XopL*_Xcc_* binds to PPI1, disrupting the early defense responses activated in *Arabidopsis* during *Xcc* infection and providing valuable insights into potential strategies for regulating PM H^+^-ATPase activity during infection. These insights shed light on the virulence strategies employed by *Xcc* and offer the potential for the development of novel control strategies against *Xcc* infections.

## 2. Results

### 2.1. Ectopic Expression of XopL_Xcc_ Inhibited PTI to Promote Xcc 8004 Proliferation in Arabidopsis

**Figure 1:**
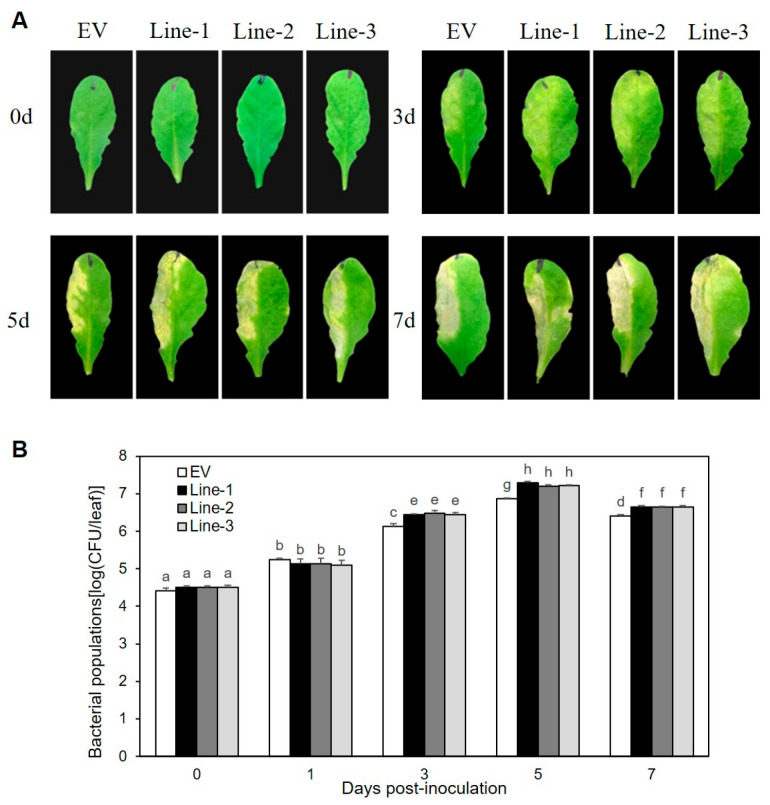
XopL*_Xcc_* promotes *Xcc* 8004 proliferation in *Arabidopsis*. (**A**) Disease symptoms. (**B**) Bacterial populations. Lines-1, -2, and -3 represent the three independent XopL*_Xcc_* transgenic plants, and EV represents the control plants. The a–g labels on panel (**B**) represent significant differences (*n* = 30, *p* < 0.05; estimated by two-way ANOVA with Tukey’s HSD test). The same letters mean no statistically significant differences.

**Figure 2:**
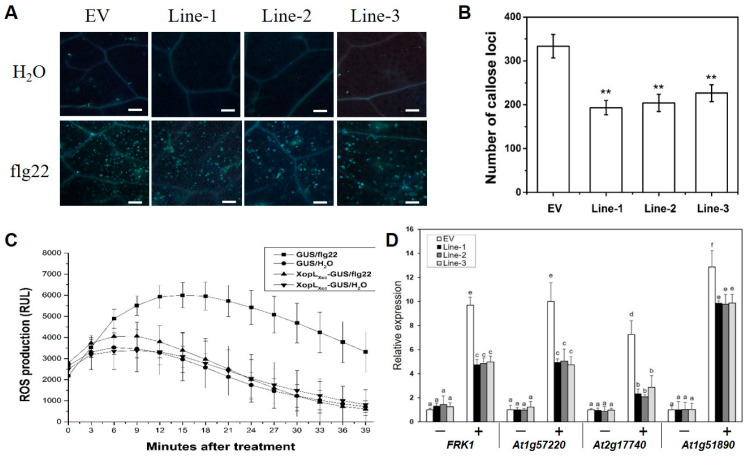
XopL*_Xcc_* intercepted pathogen-associated molecular pattern (PAMP)-triggered immunity in *Arabidopsis*. (**A**) XopL*_Xcc_* suppressed flg22-induced callose deposition. Scale bars = 0.1 mm. (**B**) Average number of callose deposits per field of view. ** *p* < 0.01 determined by Student’s *t*-test (*n* = 30). (**C**) XopL*_Xcc_* impaired flg22-induced oxidative burst. RLU, relative light units. (**D**) Transgenic expression of XopL*_Xcc_* suppressed PAMP defense response-related genes induced by Δ*hrcV*. *ΔhrcV*, T3SS-defective mutant strain. Lines-1, -2, and -3 represent the three independent XopL*_Xcc_* transgenic plants, and EV represents the control plants. The a-f labels in panel D represent significant differences (*n* = 30, *p* < 0.05; estimated by two-way ANOVA with Tukey’s HSD test). The same letters mean no statistically significant differences.

The roles of XopL*_Xcc_* in the pathogenic processes of *Xcc* 8004 were investigated by constructing three independent transgenic lines (Line1, -2, and -3) that overexpressed 35S::*XopL_Xcc_*:*GUS* (Figure S1). Upon exposure to *Xcc* 8004, all three lines exhibited more severe disease symptoms (Figure 1A) and harbored significantly larger bacterial populations compared to the control plants (Figure 1B). The flg22-induced accumulation of callose deposition (Figure 2A,B) and oxidative burst (Figure 2C) were suppressed in these lines. Additionally, the impact of XopL*_Xcc_* on disease resistance in *Arabidopsis* was assessed by analyzing the expression of four established PTI-related genes, including *FRK1*. Subsequent to inoculation with *Xcc* 8004*ΔhrcV*, their transcript levels in XopL*_Xcc_* transgenic plants were reduced by varying degrees (Figure 2D). In conclusion, these findings demonstrate that XopL*_Xcc_* suppresses plant PTI by inhibiting the expression of PTI-related genes, the generation of flg22-induced ROS, and callose deposition in *Arabidopsis*.

### 2.2. XopL_Xcc_ Interacts with PPI1 in Planta and in Yeast

**Figure 3:**
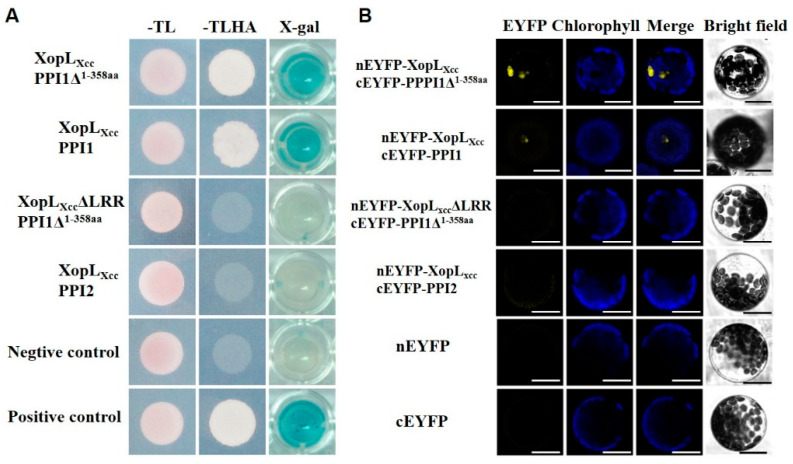
XopL*_Xcc_* interacted with PPI1 both in planta and in yeast. (**A**) Interaction of XopL*_Xcc_* with PPI1 in the split-ubiquitin-based yeast two-hybrid system. –TL, yeast growth medium lacking Trp and Leu; –TLHA, yeast growth medium lacking Trp, Leu, His, and Ade; X-gal, β-galactosidase activity of yeast transformants. (**B**) Interaction of XopL*_Xcc_* with PPI1 ascertained with a BiFC assay. Bars = 20 µm.

A yeast two-hybrid screen against a normalized *Arabidopsis* Col-0 cDNA library was conducted to identify XopL*_Xcc_* interactors (Figure S2, see Methods Section 4). The yeast strain Cub-XopL*_Xcc_* was utilized as bait, with the cDNA library serving as the prey. A total of 10^7^ primary yeast transformants were screened, resulting in the identification of 30 potential candidates. From these, PPI1, comprising 612 amino acids and encoded by *At4g27500*, was chosen due to its consistent presence during the screening process. Both the truncated protein PPI1Δ^1-358aa^ (lacking the N-terminal domain from 1 to 358 amino acids) and the full-length PPI1-encoding cDNA interacted with XopL*_Xcc_* during the yeast two-hybrid point–point verification (Figure 3A). Conversely, no interactions were observed between XopL*_Xcc_* and PPI2 (a homolog of PPI1), PPI1Δ^1-358aa^, and XopL*_Xcc_*ΔLRR (lacking LRR domains) (Figure 3A).

The BiFC assay revealed specific interactivity between cEYFP-PPI1Δ^1-358aa^ and nEYFP-XopL*_Xcc_* in *Arabidopsis*. As expected, PPI1 and XopL*_Xcc_* also interplayed with each other *in planta* (Figure 3B). Consistent with the observations in the yeast two-hybrid experiments, no interaction was evident between PPI2 and XopL*_Xcc_* or between PPI1Δ^1-358aa^ and XopL*_Xcc_* ΔLRR in the BiFC assay (Figure 3B). Together, these findings demonstrate that XopL*_Xcc_* interacts with PPI1 in plant cells. Moreover, the data suggest that XopL*_Xcc_* binds specifically to the C-terminus of PPI1, highlighting the essential role of the LRR domain in mediating this interactivity.

### 2.3. PPI1 Can Potentially Influence the Subcellular Localization of XopL_Xcc_

**Figure 4:**
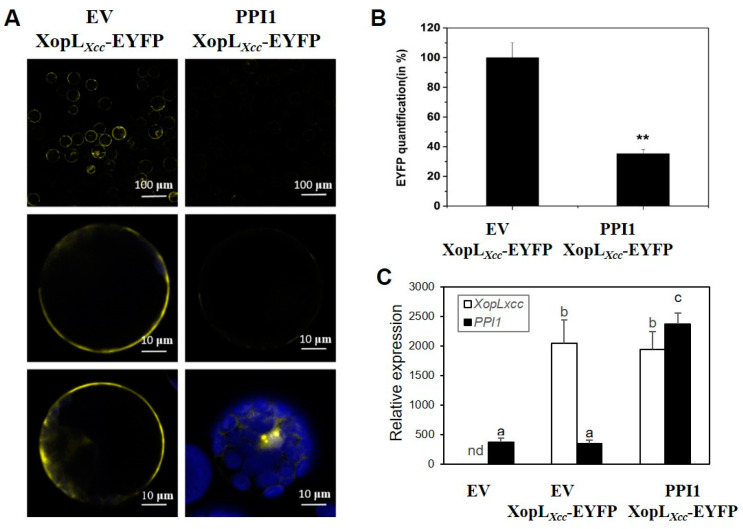
PPI1 affected the subcellular localization of XopL*_Xcc_*. (**A**) EYFP fluorescence was detected in *Arabidopsis* protoplasts co-expressing XopL*_Xcc_*-EYFP with either the EV (the left panel) or PPI1(the right panel). Row 1 displays cell fluorescence at a 100 µm scale, while Rows 2 and 3 are shown at a 10 µm scale. Both Rows 2 and 3 were subjected to identical experimental conditions, and each presents two independent typical cells. (**B**) The fluorescence intensity of EYFP at the *Arabidopsis* protoplast membrane. ** *p* < 0.01 estimated by Student’s *t*-test (*n* = 50). (**C**) The expression levels of *XopL_Xcc_* and *PPI1* in *Arabidopsis* protoplasts. The mRNA levels of all genes were normalized with *Atactin2*. The a/b/c labels represent significant differences (*n* = 30, *p* < 0.05; estimated by two-way ANOVA with Tukey’s HSD test). The same letters indicate no statistically relevant differences.

Our prior research established the subcellular localization of XopL*_Xcc_* to the cell membrane and cytoplasm [20]. However, the results from the BiFC analysis were intriguing as they demonstrated a lack of interaction between XopL*_Xcc_* and PPI1 in the plasma membrane (PM) (Figure 3B). Following this observation, we conducted transient co-expression experiments involving XopL*_Xcc_*-EYFP with either the empty vector pXSN (EV) or PPI1 in *Arabidopsis* protoplasts (Figure 4C). Under consistent fluorescence excitation and detection, significantly diminished fluorescence signals of EYFP at the PM were noted upon co-expression with PPI1 compared to EV (Figure 4A,B). These findings indicate that their interaction may have modified subcellular localization.

### 2.4. PPI1 Plays a Role in Arabidopsis Immune Response to Xcc

**Figure 5:**
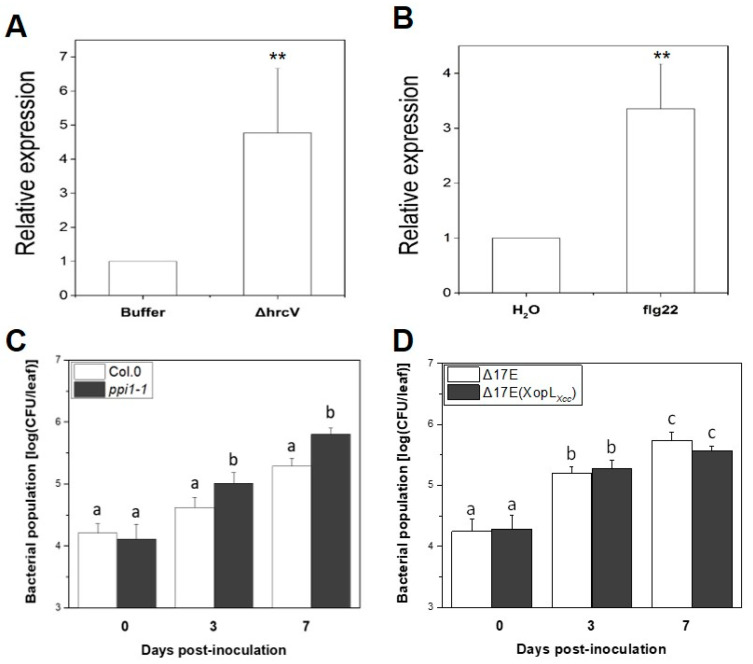
PPI1 influenced the resistance of *Arabidopsis* to *Xcc*. (**A**,**B**) The expression of *PPI1* was induced by *Xcc*Δ*hrcV* (**A**) and flg22 (**B**). Statistically significant differences at ** *p* < 0.01 were ascertained by Student’s *t*-test (*n* = 20). (**C**,**D**) Bacterial growth was assessed at 0, 3, and 7 days post-infection. The a/b/c labels represent significant differences (*n* = 30, *p* < 0.05; estimated by two-way ANOVA with Tukey’s HSD test). The same letters indicate no statistically relevant differences.

*PPI1* encodes proton pump interactor 1, which can bind to the *Arabidopsis* PM H^+^-ATPase (EC 3.6.3.6) and stimulate its activity [27]. However, the function and specific signaling mechanisms to which PPI1 responds to remain unknown. Inoculation with *Xcc* 8004Δ*hrcV* or the flg22 peptide (a 22-amino-acid sequence from the N-terminal region of flagellin) led to a 3–7-fold increase in PPI1 expression in *Arabidopsis* Col-0 (Figure 5A,B). The role of PPI1 in the response of *Arabidopsis* to *Xcc* infection was further investigated by inoculating both the wild-type Col-0 and a *PPI1* loss-of-function mutant, *ppi1-1* (SALK_042646C), with 10^6^ CFU/mL of the *Xcc* 8004Δ*hrcV* mutant (see Methods Section 4). As anticipated, *ppi1-1* exhibited markedly enhanced Δ*hrcV* bacterial growth compared to the wild type (Figure 5C). A previous study observed that XopL*_Xcc_* could amplify the pathogenicity of Δ*17E* (*Xcc* 8004 strain lacking 17 known T3Es, including XopL*_Xcc_*, as described in Table S2) in the Col-0 genotype [20]. When the same experimental procedure was applied to *ppi1-1*, no significant variations in bacterial growth were observed, as anticipated (Figure 5D). These results indicate that PPI1 potentially plays a role in the immune response of *Arabidopsis* to *Xcc* and support the hypothesis that PPI1 is a target of XopL*_Xcc_*.

### 2.5. XopL_Xcc_ Suppresses Innate Immunity in Arabidopsis by Targeting PPI1

**Figure 6:**
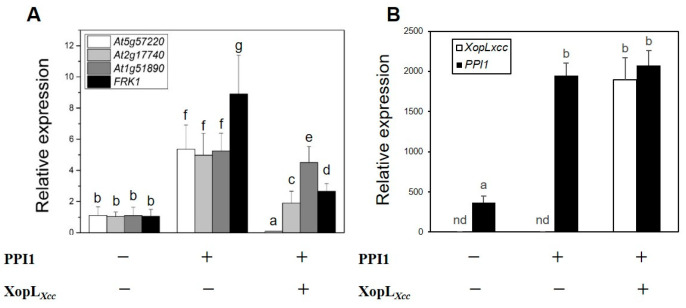
XopL*_Xcc_* suppresses the expression of PTI-related genes induced by *PPI1*. (**A**) The expression levels of PTI-related genes in *XopL_Xcc_* or *XopL_Xcc_* + *PPI1*-transfected protoplasts of *Arabidopsis* Col-0. (**B**) *XopL_Xcc_* and *PPI1* expression levels in protoplasts. The mRNA levels of all genes were normalized to those of *Atactin2*. The a–g labels represent statistically significant variations (*n* = 5, *p* < 0.05, two-way ANOVA with Tukey’s HSD test). The same letters indicate no statistically relevant differences.

In a previous investigation, XopL*_Xcc_* was demonstrated to inhibit the expression of four PTI-related genes in *Arabidopsis* protoplasts [20]. Hence, PPI1 was transiently co-expressed with XopL*_Xcc_* or empty vector in Col-0 protoplasts (Figure 6B). The results revealed that while PPI1 induced the expression of PTI-related genes by ~5–9 fold, XopL*_Xcc_* was able to suppress this response by interacting with PPI1 (Figure 6A).

**Figure 7:**
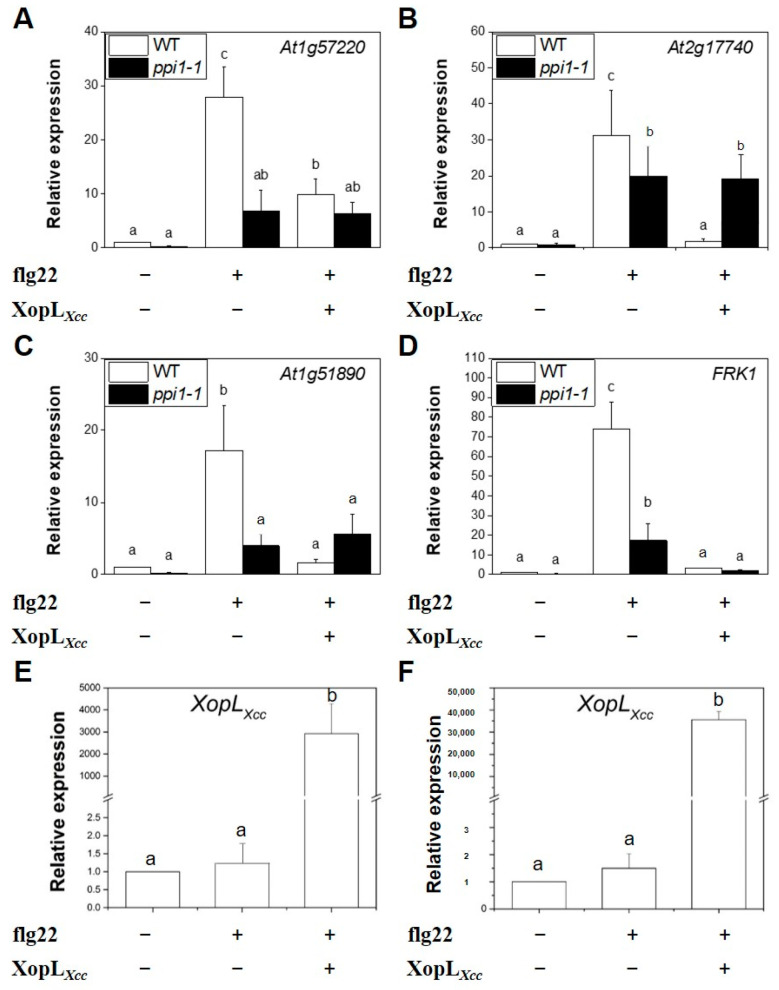
XopL*_Xcc_* could not suppress the expression of PTI-related genes in *ppi1*. (**A**–**D**) The expression levels of PTI-related genes in *XopL_Xcc_*-transfected protoplasts of *Arabidopsis* Col-0 and *ppi1-1*. (**E**,**F**) The expression levels of *XopL_Xcc_* in the protoplasts of Col-0 (**E**) and *ppi1-1* (**F**), respectively. The mRNA levels of all genes were normalized to those of *Atactin2*, and the relative expression levels were determined in protoplasts transfected with the control vector. The a/b/c labels represent statistically significant variations (*n* = 5, *p* < 0.05, two-way ANOVA with Tukey’s HSD test). The same letters indicate no statistically relevant differences.

Next, XopL*_Xcc_* was transiently expressed in Col-0 and *ppi1-1* protoplasts (Figure 7E,F). Following treatment with flg22, the expression of the four PTI-related genes in *ppi1-1* declined markedly compared to that of the wild-type Col-0 (Figure 7A–D). In Col-0, XopL*_Xcc_* suppressed the expression of PTI-related genes, which was attenuated in *ppi1-1* (Figure 7A–D). These findings underscore the importance of the interaction between PPI1 and XopL*_Xcc_* in the immune response of *Arabidopsis* to *Xcc*.

**Figure 8:**
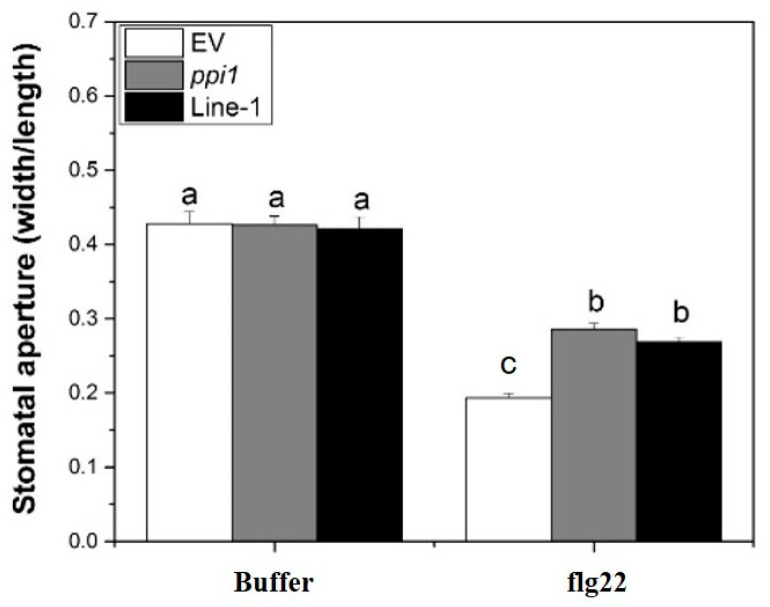
The *ppi1* mutant and XopL*_Xcc_*-transgenic plants exhibited abolished flg22-induced stomatal closure. The a/b/c labels represent statistically significant variations (*n* = 50, *p* < 0.05, two-way ANOVA with Tukey’s HSD test). The same letters indicate no statistically relevant differences.

The responses of stomatal apertures to flg22 in different lines were examined to investigate the involvement of PPI1 in flg22 signaling and stomatal immunity. The stomatal apertures of XopL*_Xcc_*-expressing lines resembled those of *ppi1-1*, exhibiting a marked increase compared to that in the control (Figure 8). In summary, these results led us to propose a model wherein XopL*_Xcc_* binds to PPI1, disrupting the early defense responses activated in *Arabidopsis* during *Xcc* infection.

## 3. Discussion

Plant pathogenic bacteria commonly secrete T3Es into host cells to modulate host responses, facilitating infection, establishment, and proliferation [1]. For instance, in *Xcc* 8004, ~12 T3Es, such as XopL, XopD, XopN, XopAC, XopK, and others, inhibited the immunity induced by flg22 in *Arabidopsis* [9]. Nevertheless, the specific functions and targets of many T3Es remain primarily unclear [32]. Given that *Xcc* 8004 is responsible for causing economically damaging black rot diseases in multiple crop species [3,4], an urgent need has arisen to identify the targets and illuminate the pathogenic mechanisms of T3Es in this specific pathogenic strain.

XopL*_Xcc_* is a member of the XopL effector superfamily, which is widespread among *Xanthomas* species and serves as a core effector group [33]. These effectors are characterized by the presence of homologs with LRR domains and an XL box known for its E3 ligase activity. XopL*_Xcv_* can suppress the expression of defense-related genes in plants, thereby undermining their immune responses. The XL box is crucial for E3 ubiquitin ligase activity and influences plastid phenotypes [33,34]. However, XopL*_Xap_* (lacking the XL box) retained the ability to suppress immune responses [22]. XopL from *X*. *euvesicatoria* (XopL*_Xe_*) directly associates with microtubules and causes severe cell death in *N*. *benthamiana* [22]. In this study, we observed that XopL*_Xcc_* suppressed innate plant immunity by reducing the expression of PTI-related genes (Figure 2D) and the generation of flg22-induced callose deposition (Figure 2A,B), as well as reactive oxygen species (ROS) (Figure 2C) in transgenic *Arabidopsis*. These results are consistent with those of prior studies conducted using protoplasts or distinct transgenic platforms [16,20].

The identification of T3E targets is a fundamental question in plant pathology [35]. Our study revealed that XopL*_Xcc_* could interact with both PPI1Δ^1-358aa^ and full-length PPI1 through yeast two-hybrid and BiFC assays (Figure 3), indicating PPI1 as one of the primary targets of XopL*_Xcc_*. Moreover, no interactivity was detected between PPI2 and XopL*_Xcc_* or PPI1Δ^1-358aa^ and XopL*_Xcc_*ΔLRR, suggesting that XopL*_Xcc_* engages explicitly with the C-terminus of PPI1, with the LRR domains being crucial for this interaction. All proteins containing LRR domains are believed to facilitate protein–protein associations [36]. Various invasive bacterial proteins were identified as containing multiple LRR domains [37]. Consequently, their absence could result in structural alterations that impact protein function.

Although the precise molecular mechanism remains elusive, our study indicates a potential role for PPI1 in the immune responses in *Arabidopsis*. Both Δ*hrcV* and flg22 could upregulate the expression of *PPI1* in Col-0 (Figure 5A,B). Notably, the expression levels of the four PTI-related genes in *ppi1-1* were significantly reduced (Figure 7A–D), aligning with the observation that *ppi1-1* exhibited markedly higher Δ*hrcV* bacterial growth compared to the wild type (Figure 5C). In *Arabidopsis* protoplasts, PPI1 induced the expression of PTI-related genes, while XopL*_Xcc_* counteracted this response through its interaction with PPI1 (Figure 6A). Notably, in Col-0 protoplasts, XopL*_Xcc_* suppressed the expression of PTI-related genes, which was mitigated in *ppi1-1* (Figure 7A–D). Moreover, the stomatal aperture of XopL*_Xcc_*-expressing lines resembled those of *ppi1-1* mutants, exhibiting a remarkable elevation compared to that of the control Col-0, which aligns with the phenotype observed in response to *Xcc* (Figure 1, Figure 2, Figure 5 and Figure 8). Thus, these findings suggest a model in which XopL*_Xcc_* binds to PPI1, disrupting the early defense responses elicited in *Arabidopsis* during *Xcc* infection.

PPI1 consists of 612 amino acids and is predicted to encode three coiled-coil regions and a transmembrane domain, which might be recruited to the PM for interaction with H^+^-ATPase [27]. Full-length PPI1 or its N-terminal domain could bind PM H^+^-ATPase at a site different from the known 14-3-3 binding locations and stimulate its activity [24]. PM H^+^-ATPase, the well-known PM H^+^ pump, is a central regulator in plant physiology, which mediates not only growth and development but also adaptation to diverse environmental stimuli [23,38,39]. Its activation can trigger immune responses [40], while its mutants exhibit a defective PAMP-triggered production of ROS, altered MAPK activation, malfunctioning PAMP-triggered stomatal closure, and changed bacterial infection phenotypes [41]. It is a crucial element in the defense mechanisms of plants against pathogen attack. However, it also functions as a target for pathogens that enable tissue invasion [42]. In *Xcc* 8004, XopL*_Xcc_* did not interact with PPI1 at the PM (Figure 4), indicating a potential inhibition of PPI1 recruitment to the PM. This hindrance could disrupt the PPI1–PM H^+^-ATPase interactivity, ultimately affecting the activation of H^+^-ATPase and immune responses in plants. In contrast, XopL*_Xcc_* downregulated the salicylic acid (SA)- and PTI-related genes (Figure 6A) [22], aligning with the enhancement in PM H^+^-ATPase activity, which could cause SA accumulation and the expression of pathogenesis-related genes in tomatoes [40]. In this context, the investigation of the possible disruption of the PPI1–H^+^-ATPase complex by XopL*_Xcc_* via ubiquitination, as well as the intricate spatial and temporal modulation of PM H^+^-ATPase activity during the initial stages of pathogen recognition, will be the emphasis of forthcoming research.

In conclusion, previous research has revealed that XopL*_Xcc_* interferes with the innate immunity of *Arabidopsis* by suppressing PTI and SA signaling, independent of MAPKs [16,20]. However, the specific virulence targets and underlying mechanisms remain incompletely elucidated. In this study, we identified proton pump interactor PPI1 as a specific virulence target of XopL*_Xcc_* in *Arabidopsis*. Moreover, the C-terminus of PPI1 and the LRR domains of XopL*_Xcc_* are pivotal for facilitating this interactivity. This novel discovery marks the first identification of PPI1’s role in conferring resistance to pathogen infection, providing valuable insights into potential strategies for regulating PM H^+^-ATPase activity during pathogen infection. These findings significantly enhance our understanding of the mechanisms employed by the T3Es of pathogenic bacteria and contribute to the development of effective strategies for controlling bacterial diseases.

## 4. Materials and Methods

### 4.1. Bacterial Strains and Growth Conditions

*Xcc* strains were cultured at 28 °C in a nutrient broth–yeast extract (NYG) medium. *Escherichia coli* and *Agrobacterium tumefaciens* strains were cultured in LB media at 37 °C and 28 °C, respectively. The antibiotics added were ampicillin (50 μg/mL), rifampicin (50 μg/mL), and kanamycin (50 μg/mL for *E*. *coli* and 25 μg/mL for *Xcc* and *A*. *tumefaciens*).

### 4.2. Vector Constructions

Full-length DNA fragments of XopL*_Xcc_*, PPI1, and PPI2 were amplified by employing *FastPfu* DNA polymerase (Beijing TransGen Biotech, Beijing, China) using the primers listed in Table S1. For transient expression in protoplasts, PCR products were cloned into the pXSN-HA vector [43]. For constructing transgenic *Arabidopsis* plants, the PCR products were cloned into the 35S::*GUS*-pBI121 vector to generate the GUS-tagged constructs.

### 4.3. Plant Growth and Generation of the Transgenic Arabidopsis Plants

The *Arabidopsis* plants were grown in a mixture of vermiculite, perlite, and peat moss (1:1:2) in an environmentally controlled growth room at 22 °C and 70% relative humidity under a 12/12 h day/night light cycle. They were transformed with *A. tumefaciens* GV3101 carrying 35S::*XopL_xcc_*:*GUS*-pBI121 or 35S::*GUS*-pBI121 using the flower-dipping method [44]. Transgenic lines were selected using 50 μg/mL kanamycin, and homozygous lines in the T_3_ generation were identified.

### 4.4. Virulence Assays, Callose Deposition Assays, and Oxidative Burst Measurement

Virulence assays of *Xcc* strains were conducted utilizing mesophyll infiltration, as previously described [39]. For the callose deposition assays, the leaves of six-week-old *Arabidopsis* plants were infused with 1 µM flg22. They were harvested 8 h after infiltration, washed with 95% ethanol, stained for callose with 0.1% aniline blue in 7 mM K_2_HPO_4_ (pH 9.5), and then mounted in 50% glycerol. They were observed using an SZX16 fluorescence microscope (Olympus, Tokyo, Japan) under ultraviolet light, and the number of callose deposits in a 0.1 mm^2^ microscopic field was counted in randomly coded samples from ten leaves by applying OpenCFU Version 1.0 software [45].

For oxidative burst measurement, the leaves of six-week-old *Arabidopsis* plants were cut into 1 mm-long strips and incubated in 200 μL of H_2_O in a 96-well plate for 12 h. Next, 1 μM flg22 in 200 μL of reaction buffer supplemented with 20 mM luminol and 1 μg of horseradish peroxidase (Sigma) was added. Luminescence was recorded for 45 min using a Synergy HT plate reader luminometer (Bio-Tek).

### 4.5. Transient Expression in Arabidopsis Protoplasts

Mesophyll protoplasts were prepared and transfected as previously described [33]. Briefly, leaves from five–six-week-old plants were used for protoplast isolation. Enzyme solutions containing Cellulase R10 and Macerozyme R10 (Yakult, Tokyo, Japan) were utilized for leaf digestion. Plasmid DNA was purified by a HiSpeed plasmid Mini kit (QIAGEN, Dusseldorf, Germany) according to the manufacturer’s instructions.

### 4.6. Gene Expression Analyses

Total RNA from the leaves or protoplasts was isolated using Trizol Reagent (Solarbio, Beijing, China). First-strand cDNA was synthesized from 500 ng of the total RNA utilizing a PrimeScript RT reagent kit (TaKaRa, Tokyo, Japan) per the manufacturer’s instructions. For real-time RT-qPCR, 20 ng of the cDNA was mixed with SYBR Premix Ex Taq (TaKaRa) and analyzed in triplicate by employing a LightCycler^®^ 480 Real-Time PCR System (Roche, Basel, Switzerland). Gene expression levels were normalized to those of the reference gene *Atactin2*. The sequences of the primers used are listed in Table S1.

### 4.7. Yeast Two-Hybrid Screening

Yeast two-hybrid assays were performed by following the *Yeast Protocols Handbook*. The leaves of four-week-old *Arabidopsis* plants were infiltrated with 10^6^ CFU/mL *Xcc* 8004Δ*hrcV*, and leaf samples were collected at 0 and 6 h. Total RNA was extracted using an RNeasy Plant Mini Kit (QIAGEN). Subsequently, reverse transcription was conducted using Switching Mechanism at 5′ End of RNA Template (SMART) technology. RT-PCR utilized the synthesized cDNA (sscDNA) as a template for dscDNA amplification. The products were purified, cleaved with S*fi*I, and ligated to the S*fi*I-digested pPR3N plasmid. Lastly, the *Arabidopsis* cDNA library was generated and employed to transform the *Escherichia coli.*

The entire XopL*_Xcc_* coding region was amplified and inserted in the pDHB1 vector to generate a fusion between the membrane protein Ost4 and the C-terminal half of ubiquitin (Cub), followed by the artificial transcription factor LexA-VP1 [46]. The yeast strain NMY51 carrying the DHB1-XopL*_Xcc_* vector was transformed with the *Arabidopsis* cDNA library. Diploid cells were selected on a medium lacking Leu, Trp, and His supplemented with 10 mM 3-aminotriazole. Then, 2 × 10^7^ transformants were screened, of which ~300 transformants that grew on the selective medium were obtained. Cells growing on the selective medium were further tested for *lacZ* reporter gene activity using a β-galactosidase assay. Direct interaction of two proteins was investigated by co-transformation of the yeast strain NMY51 with the respective plasmids; followed by the selection of transformants on a medium lacking Leu and Trp at 30 °C for 3 days; with the subsequent transfer to a medium lacking Leu, Trp, and His for growth selection; and testing of the lacZ activity in the interacting clones. To generate the PPI1 or PPI2 fusions with the N-terminal half of ubiquitin (NubG), as well as the XopL*_Xcc_* or XopL*_Xcc_*ΔLRR fusion with the C-terminal half of ubiquitin (Cub), the corresponding coding regions were amplified by PCR using the primers detailed in Table S1. They were inserted into S*fi*I sites of the pPR3N and pDHB1 vectors, respectively, and the sequence was verified.

### 4.8. Bimolecular Fluorescence Complementation (BiFC)

For the BiFC assay, XopL*_Xcc_*, XopL*_Xcc_*ΔLRR, and the candidate target genes were cloned in-frame with the EYFP fragments into the modified BiFC vectors derived from PSAT6-nEYFP-C1 or PSAT6-cEYFP-C1 [37]. *Arabidopsis* protoplasts were transfected as described previously, and the BiFC-induced YFP fluorescence was detected after 8 h by employing a TCS SP8 laser scanning confocal microscope (Leica, Solms, Germany).

### 4.9. Stomatal Aperture Measurement

The *Arabidopsis* plants were exposed to light for 2 h to ensure that most stomata were opened before treatment. Leaf peels were collected from the abaxial side of the leaves of five-week-old plants and floated in a buffer (10 mM MES [pH 6.15], 10 mM KCl, and 10 mM CaCl_2_). After treatment with 100 nM flg22 or the mock solution for 1 h, the stomata were observed under a microscope (Olympus, Tokyo, Japan). The stomatal aperture was measured by applying ImageJ version 1.0 software.

## Data Availability

Data presented in this study will be available from the corresponding author upon request.

## Supplementary Materials

The following supporting information can be downloaded at: https://www.mdpi.com/article/10.3390/ijms25179175/s1.
