# Supplementary material for: The Type III Effector XopLXcc in Xanthomonas campestris pv. campestris Targets the Proton Pump Interactor 1 and Suppresses Innate Immunity in Arabidopsis
Source: Int J Mol Sci. 2024 Aug 23;25(17):9175. doi: 10.3390/ijms25179175 (PMC11394911; doi:10.3390/ijms25179175)
Supplement: Supplementary file 1 [file ijms-25-09175-s001.zip › Supplemental Figs__Supplementary Data.pptx]

## Slide 1
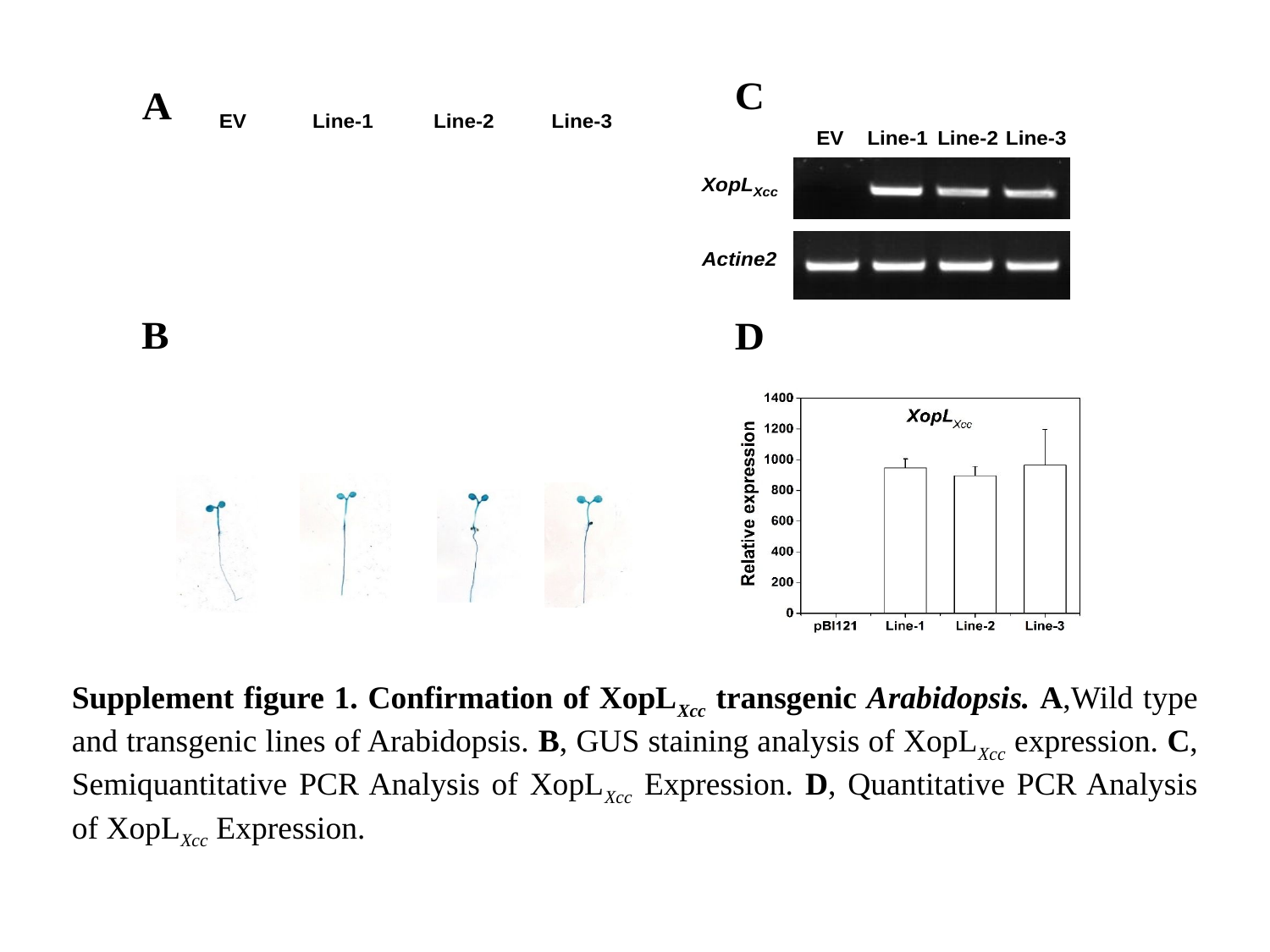

Supplement figure 1. Confirmation of XopLXcc transgenic Arabidopsis. A,Wild type and transgenic lines of Arabidopsis. B, GUS staining analysis of XopLXcc expression. C, Semiquantitative PCR Analysis of XopLXcc Expression. D, Quantitative PCR Analysis of XopLXcc Expression.

## Slide 2
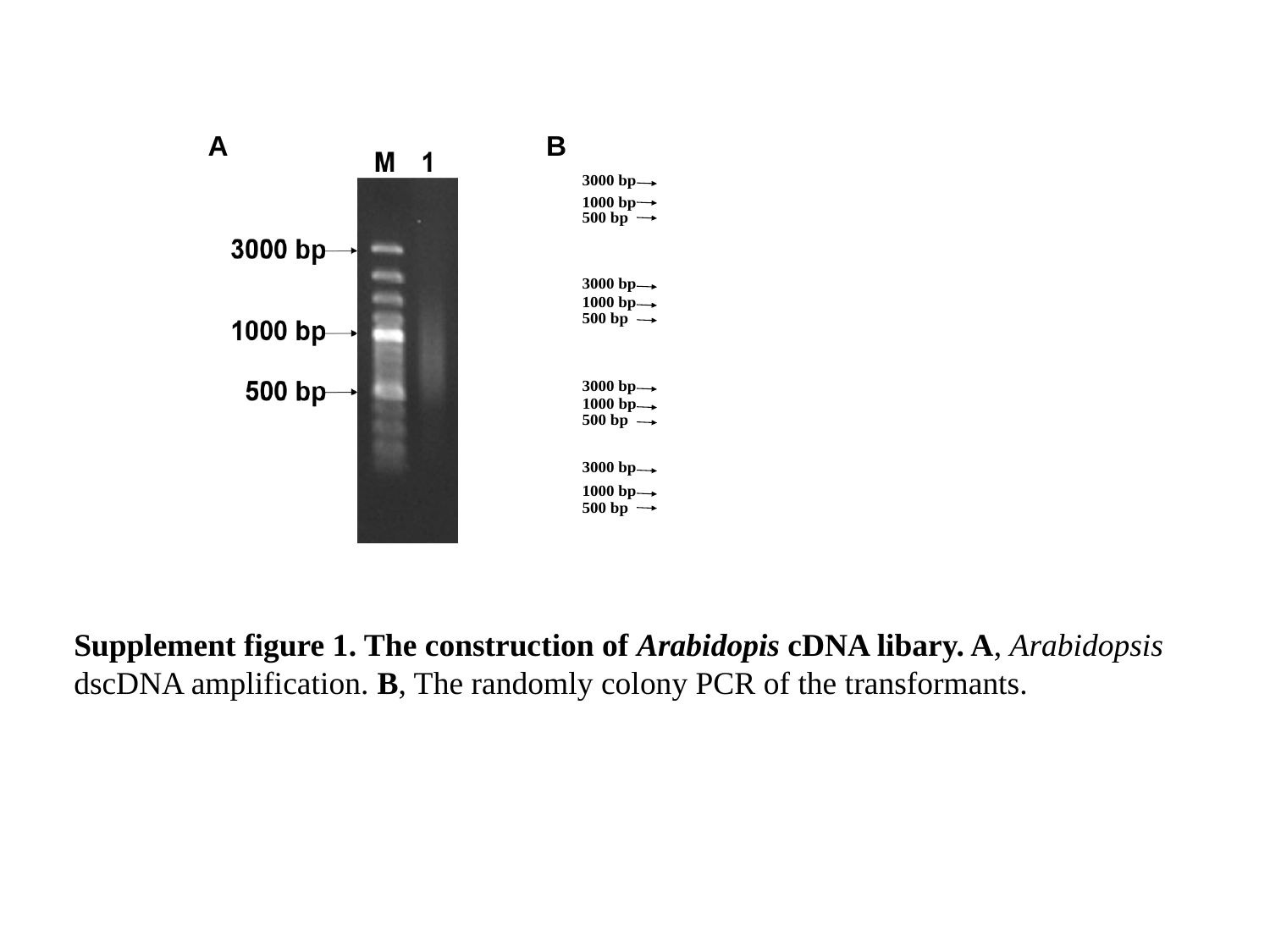

A
B
Supplement figure 1. The construction of Arabidopis cDNA libary. A, Arabidopsis dscDNA amplification. B, The randomly colony PCR of the transformants.
