# Supplementary material for: The Type III Effector XopLXcc in Xanthomonas campestris pv. campestris Targets the Proton Pump Interactor 1 and Suppresses Innate Immunity in Arabidopsis
Source: Int J Mol Sci. 2024 Aug 23;25(17):9175. doi: 10.3390/ijms25179175 (PMC11394911; doi:10.3390/ijms25179175)
Supplement: Supplementary file 1 [file ijms-25-09175-s001.zip › Table.S1_Supplementary Data.pdf]

Table S1. Primers used in this work*.

**Purpose/Category**	**Primer sequences (5'-3')**
**Name**	**Forward primers/Reverse primers**
Plasmid constructions	
XopL$_{Xcc}$-*Xho* I-F	CCGCTCGAGGTGTCAGCGGGCGGCCGGGT
XopL$_{Xcc}$-*Spe* I-R	GGACTAGTACTCTCAGAAGCGTCAGTCG
PPI1-XF	TGTATGGATGGGTGTTGAAGTTGTAAA
PPI1-XR	TGTATGGGAGAACGTAGTAGTAACCCA
PPI2-XF	TGTATGGATGAAGAGTTTGGTTTCTAAATCGG
PPI2-XR	TGTATGGCCGTAGAAGTAAAACCACAAGG
XopL$_{Xcc}$-*Sfi* I-F	GGCCATTACGGCCGTGTCAGCGGGCGGCCGGGT
XopL$_{Xcc}$- *Sfi* I-R	GGCCGAGGCGGCCCCACTCTCAGAAGCGTCAGTCG
XopL$_{Xcc}$ΔLRR-*Sfi* -R	ACGGATCGTGCAATGGTCAGGTAAGTCACTGATGGATTCGGGAAGTTC
XopL$_{Xcc}$ΔLRR-*Sfi* -F	GAACTTCCCGAATCCATCAGTGACTTACCTGACCATTGCACGATCCGT
PPI1- *Sfi* I-F	GGCCATTACGGCCATGGGTGTTGAAGTTGTAAA
PPI1- *Sfi* I-R	GGCCGAGGCGGCCCCGAGAACGTAGTAGTAACCCA
PPI2- *Sfi* I-F	GGCCATTACGGCCATGAAGAGTTTGGTTTCTAAATCGG
PPI2- *Sfi* I-R	GGCCGAGGCGGCCCCCCGTAGAAGTAAAACCACAAGG
Transgenic plants	
4273-RT-F	GTCCTGCAACGCAGTACGCA
4273-RT-R	GCAGGTCGAGCTCTTGCAGA
Atactin2-RT-F	TGTCTCGTTGTCCTCCTCAC
Atactin2-RT-R	CCTCGGTAAGAAGAACAAGG
Real-time PCR	
FRK1-QF	ATCTTCGCTTGGAGCTTCTC
FRK1-QR	TGCAGCGCAAGGACTAGAG
At1g51890-QF	CCAGTTTGTTCTGTAATACTCAGG
At1g51890-QF	CTAGCCGACTTTGGGCTATC
At2g17740-QF	TGCTCCATCTCTCTTTGTGC
At2g17740-QF	ATGCGTTGCTGAAGAAGAGG
At5g57220-QF	AATGGAGAGAGCAACACAATG
At5g57220-QR	ATACTGAGCATGAGCCCTTTG
4273-QF	AATCAACTCCTCGCCACTTAC
4273-QR	CGCAGATCAACCTCTTCCAG
PPI1-QF	CAGTATCGGATTCAACAT

PPI1-QR	TTAGCAATGACCTTATCTC
Atactin2-QF	AGTGGTCGTACAACCGGTATTGT
Atactin2-QR	GAGGAAGAGCATTCCCCTCGTA

* The Sequences underlined indicate restriction site.
