# Supplementary material for: The Type III Effector XopLXcc in Xanthomonas campestris pv. campestris Targets the Proton Pump Interactor 1 and Suppresses Innate Immunity in Arabidopsis
Source: Int J Mol Sci. 2024 Aug 23;25(17):9175. doi: 10.3390/ijms25179175 (PMC11394911; doi:10.3390/ijms25179175)
Supplement: Supplementary file 1 [file ijms-25-09175-s001.zip › Table.S2_Supplementary Data.pdf]

Table S2. Bacterial strains used in this study.

Strains or plasmids	Relevant characteristics	Reference or source
Xanthomonas campestris* pv. *campestris		
*Xcc*8004	*Xcc* strain, Wild type; Rif^r	This lab
*Xcc*8004Δ*hrcV*	Gene *hrcV* deletion mutant of *Xcc*8004; Rif^r	This lab
Xcc 8004Δ17E	17 known Type III effectors (XC0052、XC0241、XC0541、XC0542、XC1210、XC1553、XC2004、XC2081、XC2210、XC2602、XC2994、XC2995、XC3160、XC3176、XC3177、XC3802 和 XC4273) deletion mutant of 8004, Rif^r	This lab
Xcc 8004Δ17E(XopL$_{Xcc}$)	*Xcc* 8004Δ17E harboring pLAFRJ- XopL$_{Xcc}$, Rif^r, Tc^r	This lab
Agrobacterium tumefaciens		
GV3101	*A. tumefaciens* strain, Wild type; Rif^r	This lab
